# Effect of pH on the In Vitro Degradation of Borosilicate Bioactive Glass and Its Modulation by Direct Current Electric Field

**DOI:** 10.3390/ma15197015

**Published:** 2022-10-10

**Authors:** Xuanyu Zhang, Minhui Zhang, Jian Lin

**Affiliations:** 1 School of Materials Science and Engineering, Tongji University, Shanghai 201804, China; 2 Key Laboratory of Advanced Civil Engineering Materials, Ministry of Education, Tongji University, Shanghai 200092, China

**Keywords:** borosilicate bioactive glass, pH, ion release, direct current electric field, in vitro

## Abstract

Controlled ion release and mineralization of bioactive glasses are essential to their applications in bone regeneration. Tuning the chemical composition and surface structure of glasses are the primary means of achieving this goal. However, most bioactive glasses exhibit a non-linear ion release behavior. Therefore, modifying the immersion environment of glasses through external stimuli becomes an approach. In this study, the ion release and mineralization properties of a borosilicate bioactive glass were investigated in the Tris buffer and K_2_HPO_4_ solutions with different pH. The glass had a faster ion release rate at a lower pH, but the overly acidic environment was detrimental to hydroxyapatite production. Using a direct current (DC) electric field as an external stimulus, the pH of the immersion solution could be modulated within a narrow range, thereby modulating ion release from the glass. As a result, significant increases in ion release were observed after three days, and the development of porous mineralization products on the glass surface after six days. This study demonstrates the effectiveness of the DC electric field in modulating the ion release of the bioactive glass in vitro and provides a potential way to regulate the degradation of the glass in vivo.

## 1. Introduction

Bioactive glasses have attracted a lot of attention due to their wide range of potential applications in medicine, especially in bone repair [1,2,3]. Due to the formation of a bone-like hydroxyapatite (HA) layer on the surface, this kind of material can form strong bonds with bone or soft tissues [4]. Additionally, specific concentration ranges of soluble Si and Ca ions released from bioactive glasses can stimulate the expression of several genes involved in osteogenesis and angiogenesis [5,6,7,8], resulting in rapid bone regeneration. As a result, it is crucial to maintain control over the degradation of bioactive glasses to maintain a critical concentration of active ions [9].

The degradation of silicate bioactive glasses in vitro involves five reaction stages, including the rapid exchange of alkalis and alkaline earth cations with H^+^(or H_3_O^+^) from solution, the release of soluble Si ions resulting from hydrolysis of silicate networks, the formation of an Si-rich layer on the surface as a result of the condensation and repolymerization of the silanols produced in the first two stages, the growth of an amorphous CaO-P_2_O_5_-rich layer on the top of the Si-rich layer, and the crystallization of the amorphous CaO-P_2_O_5_-rich layer into an HA layer [10]. Both the properties of bioactive glasses and the external environment can influence these reactions. Since the first report of the silicate bioactive glass, 45S5 Bioglass, in the 1970s, methods for controlling the degradation rate of the glass have focused on modifying the glass’s composition or surface structure, such as varying the type or percentage of network formers [11,12], controlling the particle size [13,14], and generating nanoporous structures via the sol-gel method [15,16,17].

Borosilicate glasses have a wide range of applications for their good thermal stability, mechanical properties, and optical properties, such as solid-state lasers, laboratory glassware, display panels, and radiation shielding material [18,19,20]. In recent years, a novel class of bioactive glasses based on borosilicate, with a similar degradation mechanism to silicate bioactive glasses, has garnered considerable attention because of their more controlled and complete degradation characteristics compared to silicate bioactive glasses [11]. However, the degradation behavior of most bioactive glasses as a function of time exhibits a nonlinear profile, in which the rate of ion release is high during the early period and thereafter decreases dramatically. As a result, using external stimuli to adjust the ion release during immersion to specific requirements may be a more flexible method [21].

The pH of the solution plays an important role in the degradation of bioactive glasses. The H^+^ (or OH^−^) in the solution participates in the exchange of alkalis and alkaline earth cations and the generation of HA. In addition, the pH affects the surface charge of glasses in the solutions, which in turn affects the adsorption of ions or proteins [22,23]. Several studies have shown that the silicate bioactive glass in acidic buffer solution accelerates the Ca release and the apatite formation while decreasing the Si release [24,25,26,27]. However, there are few reports on the effect of solution pH on the degradation of borosilicate bioactive glasses [28].

Direct current (DC) electrotherapy is one of the earliest electrotherapies and has a wide range of clinical applications, including bone repair [29]. Electricity can be delivered to the site by percutaneously inserted electrodes. Numerous studies have shown that continuous direct current stimulation in the adjuvant treatment of nonunion can improve the chance of its cure [30,31,32]. The DC stimulation of osteogenesis may be related to electrochemical reactions occurring at the electrodes, such as changes in oxygen partial pressure and pH [33]. In the previous study, we found that applying a DC electric field can improve the degradation and mineralization of a borosilicate bioactive glass [34]. However, the mechanism by which the DC electric field affects the degradation of the glass is unclear.

Herein, we investigated the in vitro degradation behavior and mineralization of a melt-derived borosilicate bioactive glass in a wide range of pH values and under DC electric fields. Additionally, we explained the mechanism through which the DC electric field affected the degradation of the glass by combining the effects of pH and the DC electric field. The results are expected to provide a potential strategy for regulating the release of ions from the bioactive glass.

## 2. Materials and Methods

### 2.1. Preparation of the Glass

**Figure 1:**
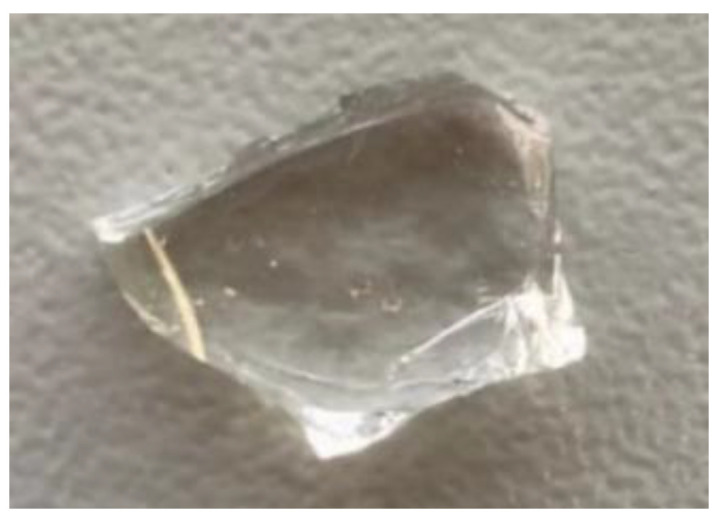
The image of the produced glass.

A borosilicate bioactive glass with the component of 18SiO_2_-36B_2_O_3_-22CaO-8MgO-8K_2_O-6Na_2_O-2P_2_O_5_ (mol%) was synthesized by the melt quenching method [11]. This glass has a lower melting temperature and is easier to prepare than silicate glasses. The conversion rate of the glass to HA is intermediate between those for silicate 13–93 and borate 13–93B3, ensuring that the glass has good bioactivity and biocompatibility [35]. Moreover, the scaffold fabricated by the glass without cytotoxicity could stimulate bone regeneration and blood vessel growth at the defect site, making it a promising bone graft material [36]. The required amounts of the raw materials were melted in a platinum crucible at 1150 °C for 1 h, and quenched on a cold stainless-steel plate to obtain the homogeneous glass. The image of the produced glass is shown in Figure 1. The glass was crushed and sieved to create particles ranging in size from 75 to 150 μm.

### 2.2. Immersion Solutions

Typically, the degradation of bioactive glasses is studied using simulated body fluid (SBF), which has ion concentrations approximate to those of human plasma. SBF was prepared according to Kokubo [37] and used to explore the degradation behavior of the glass with a DC electric field in vitro.

The effect of pH on the mineralization of the glass was studied in a K_2_HPO_4_ solution instead of SBF, as SBF is supersaturated with respect to the calcium-phosphate at physiological pH, and increasing the pH can result in precipitation [38]. K_2_HPO_4_ solutions (0.02 M) were prepared by dissolving the required amounts of K_2_HPO_4_•3H_2_O in deionized water and buffering the pH at 3, 5, 7, and 9 by adding 1 M HCl solution.

The effect of pH on the initial degradation was studied using Tris solution, devoid of inorganic ions such as Ca and P that contribute to deposition [39]. Tris buffer solutions were prepared by dissolving 6.1 g·L^−1^ of Tris in deionized water and adjusting the pH of the solutions to 2, 3, 5, 7, 8, and 9 by adding 1 M HCl solution.

### 2.3. Short-Term Degradation

The glass particles were immersed in Tris buffers of various pH with a proportion of 10 mg·mL^−1^ at 25 °C. After 1 h of immersion, the samples were removed from the solutions, washed with deionized water, and dried at 60 °C. The mass of each sample after immersion was measured using an electronic balance. The weight loss (*W*) was calculated according to the equation:

(1) $$\;W=\frac{M_{0}-M}{M}\;\times \;100\%,$$

where *M*_0_ and *M* denote the mass of the sample before and after the immersion, respectively. The pH of the solution was measured by a pH meter during the immersion. The concentration of the ions released from the glass was measured by inductively coupled plasma-atomic emission spectroscopy (ICP-AES, Optima 2100DV, Perkin Elmer, Fremont, CA, USA). After 1 day of immersion, the structure changes of the glass were analyzed by Fourier Transform Infrared Spectrometer (FTIR, EQUINOX 55, Bruker Corporation, Billerica, MA, USA). To understand the surface component of the dissolved glass, the glass pieces with dimensions of 5 × 5 × 1.5 mm were immersed in Tris buffers (initial pH = 2, 7, and 9) for 6 h (25 °C, Sa/V = 10, where Sa is the apparent surface area of the sample (mm^2^) and V is the volume of the solution (mL)) and tested by X-ray photoelectron spectroscope (XPS, Thermo ESCALAB 250XI, Thermo Fisher Scientific, Waltham, MA, USA).

### 2.4. Long-Term Degradation

The glass particles (0.35 g) were immersed in K_2_HPO_4_ solutions (35 mL) at 37 °C for 20 days. A total of 1 mL solution was extracted for the measurement of ion concentration after 1, 3, 6, 10, and 20 days for each immersion. The formation of the HA was tested by X-ray diffraction (XRD, Smartlab9, Rigaku Co., Tokyo, Japan) and FTIR after 20 days. The surface morphology of the glass after immersion was analyzed using a field emission scanning electron microscope (FESEM, Quanta 200 FEG, FEI Company, Hillsboro, OR, USA).

### 2.5. Degradation of the Glass with a DC Electric Field

**Figure 2:**
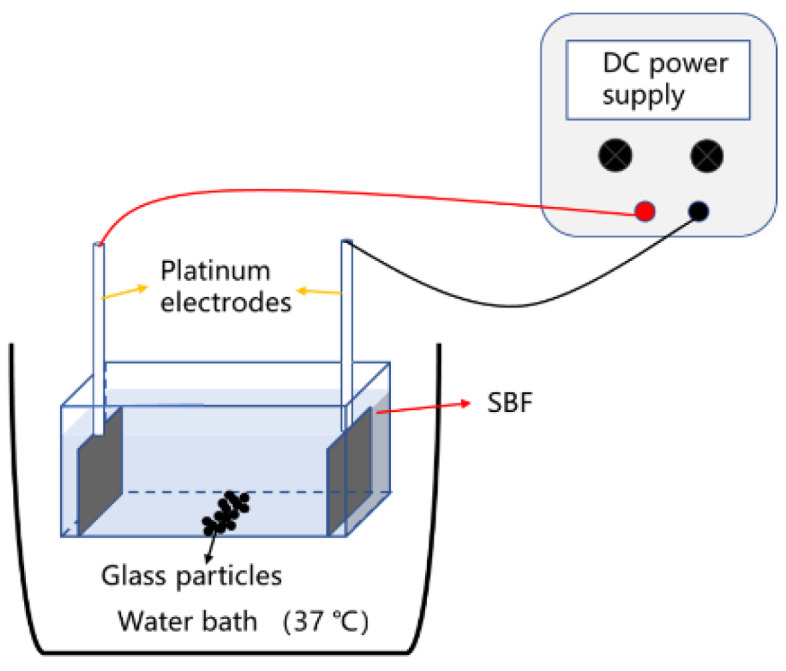
Schematic representation of the experimental setup.

The glass particles (0.2 g) were immersed in a container (4.4 × 3.2 × 2.5 cm) with SBF (20 mL) at 37 °C. Two inert platinum electrodes (20 × 15 mm) were soaked in SBF and placed on both sides of the container, through which a DC electric field was loaded using a DC adjustable power supply (PS-6402M). The particles were placed in the middle of the two electrodes and grounded. The schematic representation of the experimental setup is shown in Figure 2. The sample without a DC electric field was defined as the control group. After 1 h of immersion with a DC electric field of 30, 60, and 90 mA, the pH and ion concentration of the solution were measured after removing the sample from the solution for the measurement of weight loss. In addition, a DC electric field of 30 mA was loaded for 1 h per day for 6 days. The ion concentration of the solution was measured daily by extracting 0.5 mL of the solution. The formation of HA was tested by XRD and FTIR. The surface morphology and elemental constitution of the glass after immersion was examined by FESEM and energy dispersive spectroscopy (EDS).

## 3. Results and Discussion

### 3.1. The Effect of pH on the Degradation of the Glass

#### 3.1.1. Short-Term Degradation

The pH of the environment plays an important role in the degradation of the glass. Understanding the influence of the pH on the degradation behavior contributes to controlling the ion release from the glass. The effect of the pH on the short-term degradation of the glass was investigated in Tris solution with different pH.

**Figure 3:**
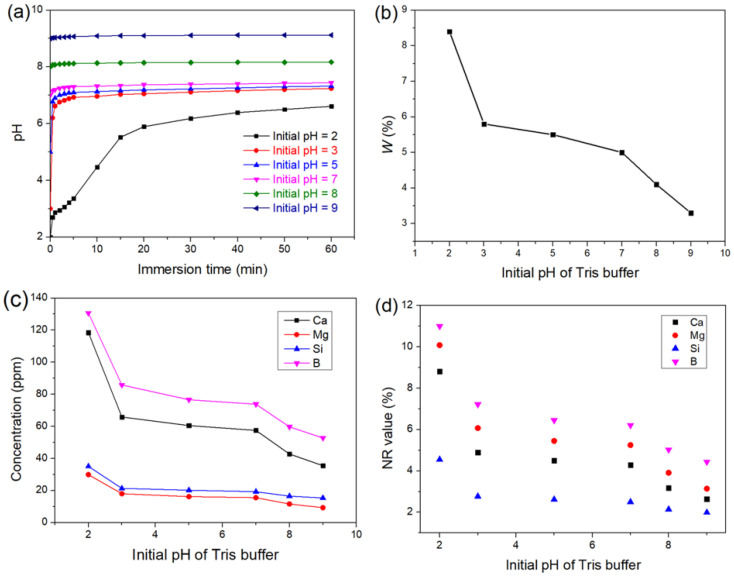
(**a**) pH variation, (**b**) weight loss, (**c**) ion release, and (**d**) NR value of the samples immersed in Tris buffers with different initial pH for 1 h.

Figure 3a shows the pH variation of the solutions versus reaction time for the glass samples immersed in Tris buffers with different initial pH for 1 h. For each immersion, the pH value increased with immersion time due to the consumption of H^+^ by the dissolution of Na^+^, K^+^, Ca^2+^, and Mg^2+^ on the glass surface. The pH changed significantly within the first minute of immersion due to the particles dissolving rapidly as they mixed with the solutions. In the acidic solutions (pH = 2, 3, and 5), the changes in pH were more pronounced due to the Tris solution’s low buffering capacity at pH < 7. In the pH = 7 solution, the pH rose to 7.4 within 1 h, whereas the pH changes in the pH = 8 and pH = 9 solutions were much smaller, only reaching 0.16 and 0.12, respectively, indicating that the pH = 7 solution contained higher concentrations of alkalis and alkaline earth cations released from the glass.

Figure 3b shows the weight loss of the samples versus the initial pH of Tris solutions within 1 h. The weight loss decreased with the increase in the initial pH of the solutions, suggesting that the glass had poor acid resistance. In the Tris solutions with buffering capacity, the weight loss of the sample varied significantly and approximately linearly with pH. In the solutions (pH = 2, 3, and 5) beyond the buffering capacity of Tris, a minor difference in weight loss was observed between samples immersed in the pH = 3 and pH = 5 solutions, while the weight loss of the sample in the pH = 2 solution increased significantly and was nearly 1.4 times that of the sample in the pH = 3 solution. The poor acid resistance of the glass is attributed to the high concentration of alkalis and alkaline earth cations, which act as network modifiers and lead to the formation of the non-bridge oxygen (NBO) groups, disrupting the continuity of the glassy network. The abundant presence of non-bridge oxygen groups favors rapid ion exchange and plays a key role in the dissolution of the glassy network [40]. Additionally, the high concentration of B in the glass prevents it from completely forming a three-dimensional network, leading to lower chemical durability. The low hydrolysis resistance of Si–O–B also contributes to the relatively low acid resistance of the glass [41].

Figure 3c shows the impact of the initial pH on the release of B, Si, Ca, and Mg. The release of the elements reduced as the initial pH of the solutions increased, similarly to weight loss. The Ca concentration increased significantly with decreasing pH, especially in the pH = 2 solution, indicating that Ca release is sensitive to the pH of the solution. Increased release of Ca at lower pH was observed in both silicate and borosilicate bioactive glasses [24,27,28], which is consistent with the mechanism by which Ca dissolves from the glass ((Si–O)_2_Ca + 2H^+^ → 2Si–OH + Ca^2+^ or (B–O)_2_Ca + 2H^+^ → 2B–OH + Ca^2+^). The release of B increased in acidic solutions, due to electrophilic attack by protons on Si-O-B bonds [42], which also resulted in an increase in Si release. In contrast, silicate bioactive glasses generally showed faster Si release at higher pH, because of the promoted nucleophilic attack of the silicate network [24]. However, it should be emphasized that the release of Si did not always increase with a drop in pH and might exhibit a minimum near neutral pH in borosilicate glasses [43,44]. The pH-dependent release of Si varied with glass composition [45], such as SiO_2_/B_2_O_3_ ratio. In our study, the results indicate that the increased H^+^ concentration accelerates the ion exchange of alkalis and alkaline earth cations, exposing the glass network to erosion by water and increasing the dissolution of the network formers, such as B and Si.

Figure 3d shows the normalized release (NR) of Ca, Mg, B, and Si, which represents the ratio of the release of the element to its content in the glass and helps to understand the extent of the degradation of the glass. NR value can be calculated according to the following equation:

(2) $${\mathrm{NR}}_{i}=\frac{C_{i}V}{{Mw}_{i}}\;\times \;100\%,$$

where *C_i_* is the mass concentration of element *i* in the solution, *V* is the volume of the solution, *M* is the mass of the sample before immersion, and *w_i_* is the mass fraction of element *i* in the glass. The NR values of the elements were different for each immersion, indicating that the dissolution of the glass was not uniform. The NR values of Ca were lower than that of Mg because Ca^2+^ was more likely to deposit due to the low solubility of calcium phosphate (CaP). For each immersion, the NR value of B was significantly greater than that of Si, being more than twice as high, suggesting the formation of a Si-rich layer on the surface.

**Figure 4:**
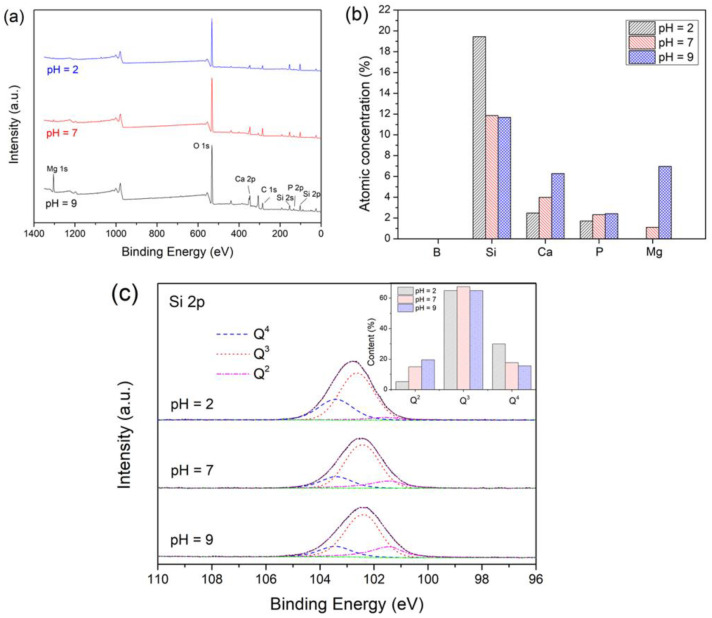
(**a**) XPS spectra, (**b**) representative atomic concentrations, (**c**) Si 2p spectra of the glass samples after immersion in Tris buffers with different initial pH for 6 h.

Figure 4a shows the XPS spectra of the samples after immersion in Tris buffers with different initial pH for 6 h, while the representative atomic concentrations of the studied glass samples are shown in Figure 4b. After each immersion, the surface was free of B, while a high concentration of Si was observed, suggesting that a Si-rich layer was generated on the surface, which was consistent with the previous study [46]. In lower pH solutions, the ion exchange and hydrolysis of the glass network were more rapid, resulting in more silanols on the glass surface, which facilitated the formation of an Si-rich layer, and consequently, a higher Si concentration on the surface. The concentration of Ca and P increased at higher pH, the reasons for which were as follows: (1) The ions were released more rapidly at lower pH; (2) CaP was more easily generated at higher pH due to its lower solubility. Likewise, the Mg concentration on the surface of the glass immersed in the pH = 9 solution was significantly higher than that in the pH = 7 solution. This might be related to the additional Mg deposition, which was detrimental to further ion release from the glass.

Figure 4c shows the Si 2p spectra of the samples. The electron density over the central, tetrahedrally coordinated Si atom is affected by the number of bridging and non-bridging oxygen atoms bonded to it, thus leading to the various binding energy of the Si 2p peaks [47]. The higher the number of non-bridging atoms bonded to a Si atom, the greater the electron density over it, and the lower the binding energy of the peak. The Si 2p spectra of all the samples were fitted with three peaks. The peak positions appear to be essentially the same (103.4/103.4/103.4 eV, 102.6/102.4/102.4 eV, and 101.6/101.4/101.4 eV for the samples immersed in the initial pH = 2/7/9 solutions, respectively). For each sample, the peaks can be assigned to Q^4^, Q^3^, and Q^2^ (Q^n^, where Q represents the Si tetrahedral unit and n is the number of bridging oxygen per tetrahedral) Si species in decreasing order of binding energy, respectively [48,49]. The results indicated a significantly depolymerized silicate layer with a high concentration of non-bridging groups at each sample surface [50]. The inset shows the content of the Q species for the samples. The decrease in the initial pH of the Tris buffers resulted in a decrease in the content of Q^2^ species and an increase in the content of Q^4^ species. The results indicated that the lower pH environment promoted the generation of a silica gel layer with a higher degree of polymerization.

**Figure 5:**
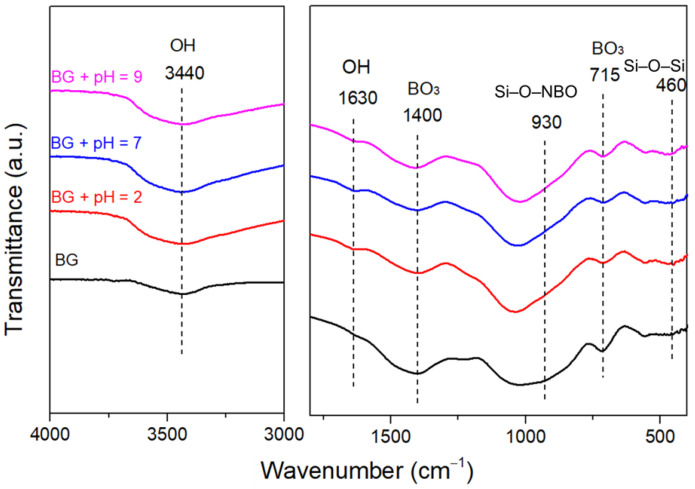
FTIR spectra of the glass samples immersed in Tris buffers with different initial pH for 1 day.

Figure 5 shows the FTIR spectra of the glass samples before and after 1 day of immersion in Tris solutions with varying initial pH. The peak around 460 cm^−1^ was assigned to the bending vibration of Si–O–Si, whereas the peaks around 715 and 1400 cm^−1^ were assigned to the bending vibration and antisymmetric stretching vibration of [BO_3_], respectively [51]. The transformation of [BO_3_] into [BO_4_] was confirmed by the presence of a broad band between 800 and 1200 cm^−1^, which was mainly assigned to the stretching vibration of [BO_4_] [52,53]. The broad band was also composed of the antisymmetric stretching vibration of Si–O–Si, and the vibration mode of non-bridging oxygen groups and Si–O–B [54]. After immersion, the bending and stretching vibration peaks of O–H appeared significantly at 1630 and 3440 cm^−1^, respectively, which were related to the ion exchange of alkalis and alkaline earth cations and the formation of a hydrated Si-rich layer [55]. The shoulder at around 930 cm^−1^ assigned to the vibration of Si–O–M (where M are modifying ions) disappeared due to the hydrolysis of the network that accompanied the ion exchange of metal cations [55,56]. The decrease in the B–O bending vibration peak and antisymmetric stretching peak in [BO_3_] was due to the rapid hydrolysis of the network, which was consistent with the rapid release of B shown in Figure 3d.

#### 3.1.2. Long-Term Degradation

The formation of HA has an essential impact on the bioactivity of the glass. Moreover, the formation of deposits changes the ion concentration and hinders the dissolution of the glass. The effect of pH on the formation of HA was investigated by immersing the glass in the K_2_HPO_4_ solution with different pH, while the changes in the ion concentration were also measured.

**Figure 6:**
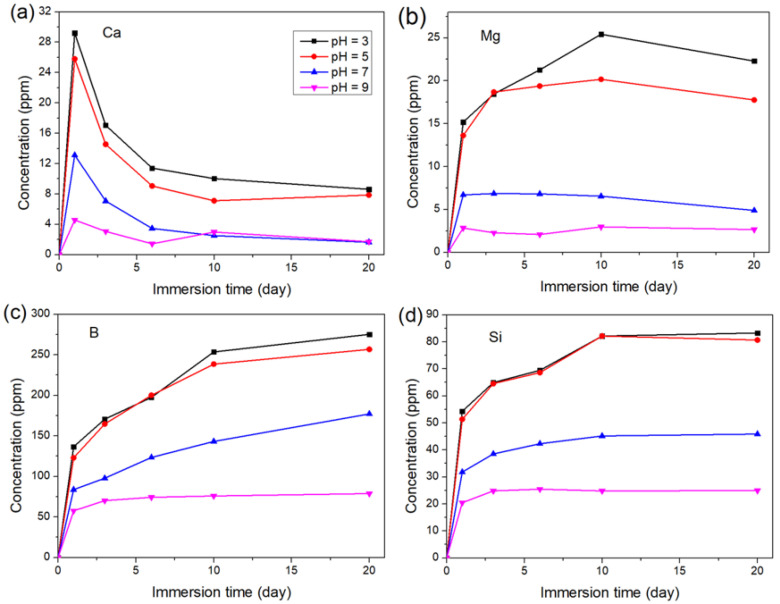
Time-dependent release of (**a**) Ca, (**b**) Mg, (**c**) B, and (**d**) Si of the glass samples in K_2_HPO_4_ solutions with different initial pH.

Figure 6 shows the time-dependent release of Ca, Mg, B, and Si from the glass samples in K_2_HPO_4_ solutions with different initial pH. The curves were labeled by the initial pH of the solutions. In Figure 6a, the concentration of Ca for each immersion reached a maximum on the first day and then decreased, indicating that CaP had been generated on the glass surface. After 6 days, the Ca concentration varied slightly due to equilibrium between Ca release and deposition. The Ca concentration decreased as the solution pH increased because the solubility of CaP is lower at a higher pH. In Figure 6b, a decrease in the Mg concentration was observed in each solution, indicating that Mg was deposited on the surface. The precipitation of Mg was later than that of Ca, especially in lower pH solutions, because the solubility of magnesium phosphate is higher than that of calcium phosphate. The deposition of Mg began at a lower concentration and earlier as the solution pH increased due to the higher concentration of PO_4_^3−^ in the solution with higher pH. The result supports the analysis of the Mg concentration on the glass surface after a short-time immersion in the solutions with different pH (Figure 4b). In Figure 6c, The B concentration increased rapidly on the first day, followed by a decrease in the release rate. The release of B was more rapid at lower pH, which was consistent with the findings in the previous section. The Si release curve followed a similar pattern, as shown in Figure 6d. Despite mineralization products on the glass surface impeding the degradation of the glass, the sustained ion release showed significant differences in various pH solutions, indicating the feasibility of regulating the degradation of the glass by modulating the pH of the environment.

**Figure 7:**
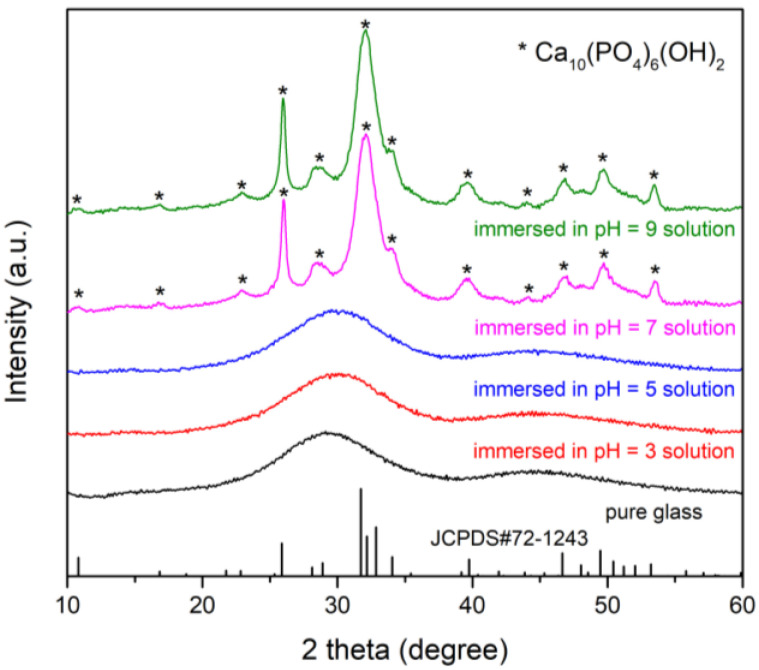
XRD patterns of the glass samples before and after immersion in K_2_HPO_4_ solutions with different initial pH for 20 days.

XRD patterns of the glass samples before and after immersion in K_2_HPO_4_ solutions with different initial pH for 20 days are shown in Figure 7. The pattern of the pure glass was absent of crystalline peaks, confirming the amorphous nature of the glass before immersion. The patterns of the glass immersed in the pH = 7 and pH = 9 solutions were characterized by distinct diffraction peaks corresponding to those of hydroxyapatite (JCPDS#72-1243). The width of the diffraction peaks indicated that the hydroxyapatite generated was either poorly crystalline or nanometer-sized, or both. The patterns of the samples immersed in the pH = 3 and pH = 5 solutions exhibited typical amorphous characteristics, suggesting that only a trace amount of hydroxyapatite was generated. Although amorphous calcium phosphate (ACP) was produced in lower pH solutions, few of them crystallized due to the low concentration of OH^−^.

**Figure 8:**
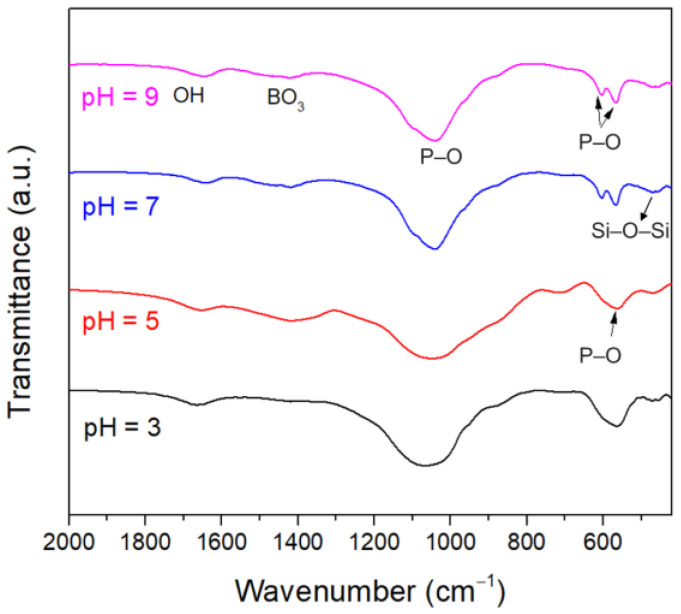
FTIR spectra of the glass samples immersed in K_2_HPO_4_ solutions with different initial pH for 20 days.

Figure 8 shows the FTIR spectra of the glass samples immersed in K_2_HPO_4_ solutions with different initial pH for 20 days. The peaks corresponding to the P–O bending vibration in crystalline HA were observed at 568 and 605 cm^−^^1^ [57] for the samples immersed in the pH = 7 and pH = 9 solutions. In contrast, the P–O bending vibration associated with the formation of the ACP was characterized by a peak around 560 cm^−1^ [10] for the samples immersed in the pH = 3 and pH = 5 solutions, indicating that crystallization did not proceed. The results were consistent with the XRD patterns.

**Figure 9:**
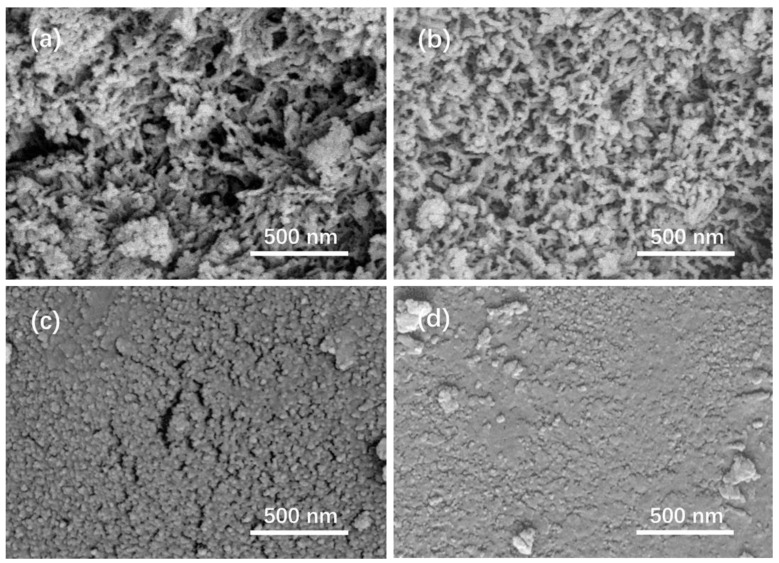
SEM image of the glass samples after immersion in K_2_HPO_4_ solutions with (**a**) initial pH = 9, (**b**) initial pH = 7, (**c**) initial pH = 5, and (**d**) initial pH = 3 for 20 days.

Figure 9 shows SEM images of the surfaces of the glass samples after immersion in K_2_HPO_4_ solutions with different initial pH for 20 days. For the glass samples immersed in pH = 9 and pH = 7 solutions, the surfaces were highly porous and composed of rod-like crystals, characteristic of HA, with diameters of about 40 nm. In contrast, the surfaces of the glass samples immersed in pH = 5 and pH = 3 solutions were dense and relatively smooth, consisting of amorphous spherules with diameters of about 30 nm. The distinction in morphology was attributed to the different products, which were confirmed by XRD and FTIR.

Table 1 summarizes the degradation of the glass in Tris and K_2_HPO_4_ solutions with different initial pH. According to the results, the ion exchange and hydrolysis of the glassy network proceeded faster in the lower pH environment, resulting in faster ion release. The concentration of Ca was limited in K_2_HPO_4_ solutions with different pH for the reaction with PO_4_^3−^ to form a CaP layer on the glass surface. However, the crystallization of HA could be inhibited in a more acidic environment. These are the basis for regulating the degradation of the glass by adjusting the pH.

### 3.2. The Degradation of the Glass with a DC Electric Field

It is well known that the pH of the electrolyte changes due to the electrolysis that occurs at the electrodes when a DC electric field is applied. The effect is utilized in the treatment of nonunion by DC electrotherapy. In this study, DC fields were loaded in the SBF, and a decrease in pH could be observed. As a result, the degradation of the glass was controlled. The magnitude of the change in pH is related to the position relative to the electrodes during electric field loading. Therefore, the samples were placed uniformly in the middle of the two electrodes to reduce this effect.

**Figure 10:**
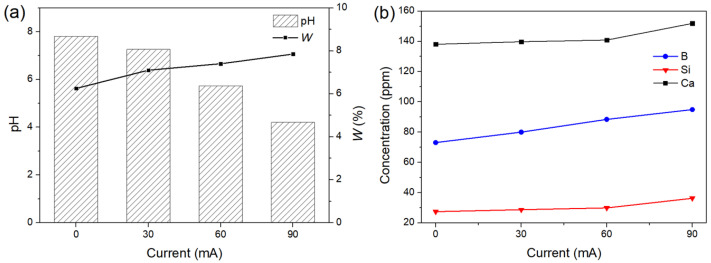
(**a**) The solution pH, weight loss, and (**b**) ion release after applying DC electric fields of varying current strengths for 1 h.

The solution pH, weight loss, and ion release after applying DC electric fields of varying current strengths for 1 h are shown in Figure 10. To generate sufficient pH gradients, currents of 30, 60, and 90 mA were used. Changes in pH were caused by a combination of glass degradation and electrolysis, which raised and reduced the pH of SBF, respectively. The solution pH decreased with increasing current intensity because the more intense electrolysis caused by the larger current resulted in a greater change in pH, which could not be entirely neutralized by the glass degradation. The weight loss of the samples increased with a greater reduction in pH caused by larger currents, which was consistent with the previously discussed effect of pH on the degradation of the glass. The ion release showed a similar trend and significantly increased at the 90 mA point due to the more acidic environment. The results indicate that it is feasible to control the degradation of the glass by modulating the solution pH using a DC electric field.

**Figure 11:**
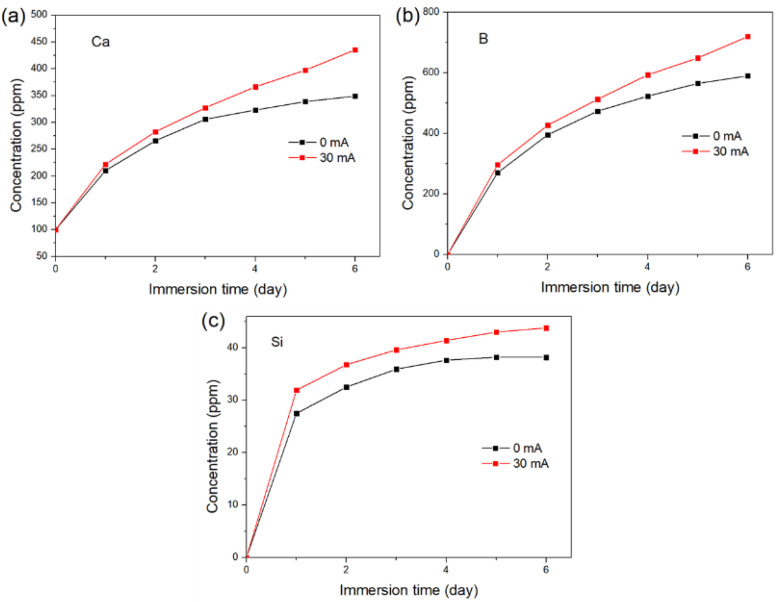
Time-dependent release of (**a**) Ca, (**b**) B, and (**c**) Si from the glass samples with and without a DC electric field applied for 6 days.

Figure 11 shows the time-dependent release of Ca, B, and Si from the glass samples with and without a DC electric field applied for 6 days. The electric field was applied for 1 h per day with an intensity of 30 mA, and the pH of the solution was controlled to be not less than 7.2. As shown in Figure 11a, both samples exhibited rapid Ca release on the first day, followed by continuous Ca release at a constant rate for the sample with an applied electric field and gradually decreasing Ca release for the control sample. The difference in the rate of Ca release between the two samples was more noticeable after 3 days. Similar patterns were observed in the B release, as shown in Figure 11b. In Figure 11c, the concentration of Si in the control group had stabilized after 5 days, whereas the sample with the applied electric field continued to have a positive Si release. The results further demonstrate that the DC electric field can regulate the ion release of the bioactive glass.

**Figure 12:**
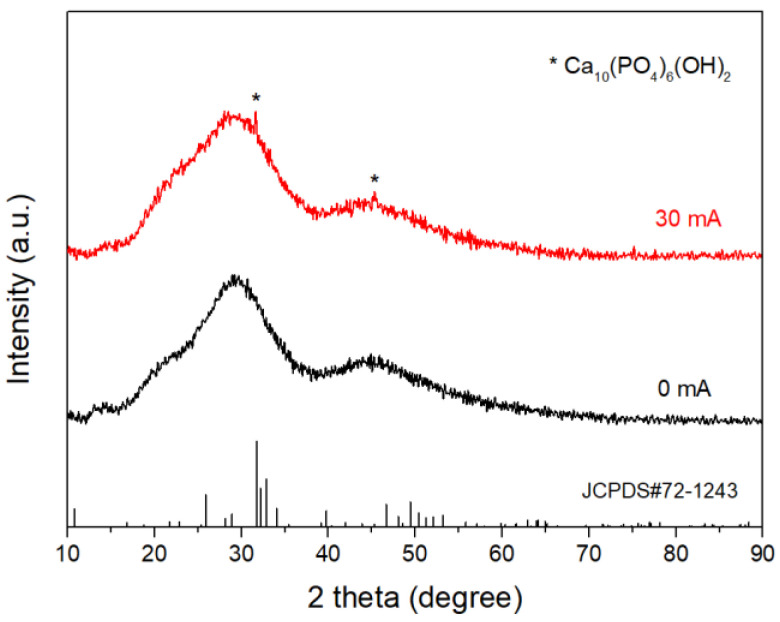
XRD patterns of the glass samples after immersion in SBF for 6 days.

Figure 12 shows the XRD patterns of the samples immersed in the SBF for 6 days. Both samples were still mainly composed of amorphous phases after immersion in SBF. However, two weak peaks corresponding to those of HA (JCPDS#72-1243) were detected for the sample with the DC electric field, confirming the formation of HA layer on the surface.

**Figure 13:**
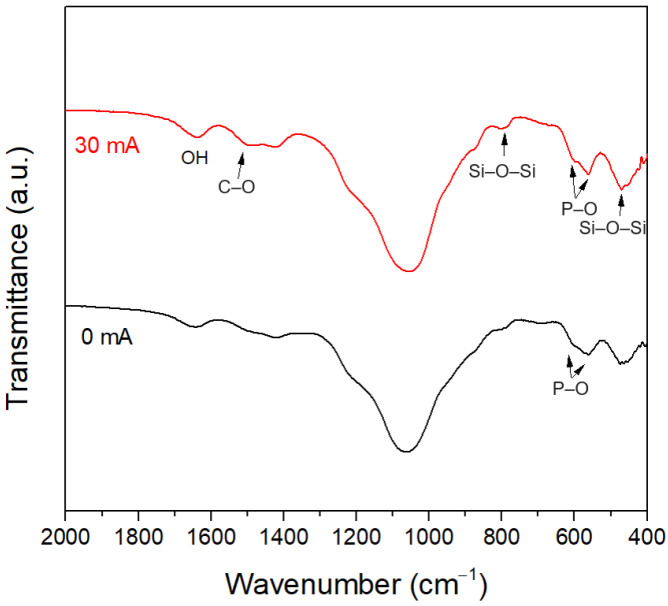
FTIR spectra of the glass samples immersed in SBF for 6 days.

The HA formation of the glass samples after immersion in SBF for 6 days was further analyzed by FTIR spectra, as shown in Figure 13. For the sample without the DC electric field, the P–O bending vibration tended to separate into the two modes, indicating that HA began to crystallize on the surface. For the sample with the DC electric field, the two P–O peaks characteristic of crystalline HA were more distinct, indicating that HA crystallized faster with the DC electric field. In addition, the peak around 1490 cm^−1^ was assigned to the C–O antisymmetric stretching vibration in the CO_3_^2−^ group [55], indicating that carbonate-substituted HA was formed.

**Figure 14:**
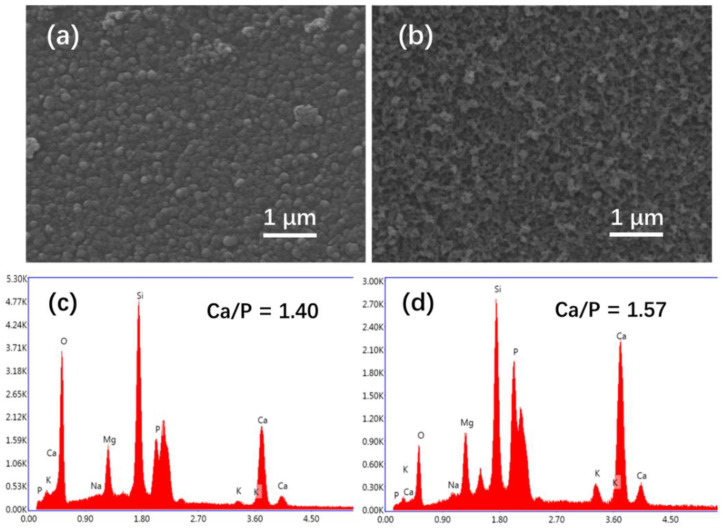
SEM image of the glass samples immersed in SBF for 6 days (**a**) without and (**b**) with a DC electric field; EDS analysis for the glass samples immersed in SBF for 6 days (**c**) without and (**d**) with a DC electric field.

SEM images of the glass samples immersed in SBF for 6 days are shown in Figure 14. After 6 days of immersion, the mineralization products covered the surface of the glass samples. For the sample with an applied electric field, the products exhibited a sparse and porous structure composed of nano-sized rod-like crystals (Figure 14b), similar to the morphology of HA formed on the surfaces of the glass after immersion in K_2_HPO_4_ solutions for 20 days (Figure 9a,b). Similar morphology was also observed on the 13-93B3 after immersion in SBF for 7 days [58]. In contrast, the control sample contained spherical nanoparticles that were more densely packed (Figure 14a), similar to the surface morphology of the 13–93B3 after immersion in SBF for 3 days [58]. Such transformation of the products from spherical particles to rod-like crystals over time was also observed in some other studies [59,60,61,62]. The EDS analysis (Figure 14c,d) showed that the glass surface was rich in Ca and P, confirming the formation of CaP layer on the glass surface. Compared to the glass samples without a DC electric field, the glass samples with a DC electric field had a higher Ca/P atomic ratio, lower than the value for stoichiometric HA (1.67), indicating a calcium-deficient apatite layer on the surface [53]. The EDS analysis was consistent with the FTIR spectra and XRD pattern that demonstrated the formation of HA on the glass samples with a DC electric field. In SBF, the HA on the glass surface was formed via ACP, as shown in Equation (3).

(3) $${\;3\mathrm{Ca}}_{3}{{(\mathrm{PO}}_{4})}_{2}\left(S\right){+\mathrm{Ca}}^{2+}{+2H}_{2}O\;\to {\;2\mathrm{Ca}}_{5}{{(\mathrm{PO}}_{4})}_{3}\left(\mathrm{OH}\right)\left(S\right){+2H}^{+}$$

The formation rate of HA was dependent on the concentration of Ca in the SBF. The higher Ca concentration in the vicinity of the sample with a DC electric field increased the driving force for ACP converting to HA, leading to the faster nucleation and growth of HA, which explained the higher Ca/P ratio of the glass with a DC electric field and difference in morphology.

The present results suggest that application of DC electric fields could provide a method for controlling the in vitro degradation of borosilicate bioactive glasses. In some applications, bioactive glasses need to be pre-treated before implantation to avoid the development of possible pH-dependent cytotoxicity due to significant changes in localized pH caused by rapid ion exchange in the early stage [63]. The application of DC electric fields may allow for more efficient pretreatment without reducing bioactivity. In addition, DC electric fields may provide a potential method for regulating the in vivo degradation of bioactive glasses. In this case, current parameters would be different from those used in vitro and require more caution. In animal tissues, pH changes occur around electrodes and are sensitive to current [64,65]. To avoid extreme changes in pH, current intensity must be less than that in vitro. Electrical current administered to bone are generally between 5 and 100 μA [66]. Regulating the in vivo degradation of bioactive glass by DC electric fields needs further investigation.

## 4. Conclusions

In this study, we have investigated the degradation behavior of a borosilicate bioactive glass in solutions with varying pH values. The glass contains a large number of non-bridging oxygen groups and B–O groups, which gives it relatively low acid resistance and a greater degradation rate in environments with lower pH, as reflected by a faster ion release. The lower pH environment retards the deposition of Mg on the glass surface and reduces ion release impediments. However, a more acidic environment can hinder the crystallization of hydroxyapatite. According to the above conclusions, the increase in solution pH caused by glass degradation is detrimental to its further degradation. DC electric fields can change the pH of SBF by electrolysis, and a larger current causes a greater change. Therefore, using DC electric fields with appropriate current intensity can modulate the solution pH in the physiological range, resulting in faster ion release and HA growth from the glass surface. This work may provide a potential method for regulating the degradation of bioactive glass in vivo.

## Figures and Tables

**Table 1:** Results of ion release in Tris (1 h) and K_2_HPO_4_ (1 d) solutions with different initial pH and product in K_2_HPO_4_ solutions (20 d) with different initial pH.

Media	Initial pH	Ion Release (ppm)				Product
		B	Si	Ca	Mg	
Tris	2	130.7	35.1	118.5	29.9	-
Tris	3	85.8	21.4	65.8	18.0	-
Tris	5	76.6	20.2	60.5	16.2	-
Tris	7	73.8	19.2	57.5	15.6	-
Tris	8	59.7	16.5	42.8	11.6	-
Tris	9	52.8	15.4	35.5	9.3	-
K_2_HPO_4_	3	136.6	54.3	29.2	15.2	ACP
K_2_HPO_4_	5	123.0	51.3	25.8	13.6	ACP
K_2_HPO_4_	7	83.8	31.8	13.1	6.7	HA
K_2_HPO_4_	9	57.6	20.5	4.6	2.8	HA

## Data Availability

The data presented in the study are available on request from the corresponding author.
